# Community support for caring relatives of people with dementia: qualitative analysis using the Theoretical Domains Framework

**DOI:** 10.1007/s10389-022-01744-w

**Published:** 2022-08-12

**Authors:** Maren Wittek, Henrike Voß, Anna Kiefer, Stefanie Wiloth, Eric Schmitt

**Affiliations:** Institute of Gerontology, Ruprecht-Karls-University of Heidelberg, Bergheimer Straße 20, 69115 Heidelberg, Germany

**Keywords:** Caring relatives, People with dementia, Municipal community, Support services, Theoretical Domains Framework, Implementation

## Abstract

**Aim:**

Although caring relatives of people with dementia are a mainstay of many care systems, the availability of support services for them within the municipal community shows deficiencies. Adopting the Theoretical Domains Framework (TDF) this study aims to investigate 1) which of the TDF domains adapted to gerontology show up in public dialogue, and 2) the results that public dialogues produce in terms of support services for caring relatives.

**Subject and methods:**

The data consists of town hall meetings and focus groups from 14 municipal communities in Germany. Participants were caring relatives and stakeholders of the communities. A qualitative content analysis was conducted, focusing on the assessment of three TDF domains, namely *knowledge, goals*, and *sociopolitical context* as well as outcomes of care optimisation.

**Results:**

With regard to domain *knowledge*, it was evident that in every community there were actors aware of the situation and relevance of carers and their relatives. Only some actors mentioned *goals* for optimising the care of the target group. The *sociopolitical context* is often addressed through statements about incomplete requirements.

**Conclusion:**

Overall, a relation between the discussion about the domains in public dialogues and changes in supporting carers of people with dementia can be assumed. The results indicate that an increased discussion about the domains within town hall meetings influences the actors and their statements with regard to the improvement of support services for caring relatives of people with dementia. Since the domains were not developed exclusively for the outlined context, this approach can also be applied to other areas of care.

**Supplementary information:**

The online version contains supplementary material available at 10.1007/s10389-022-01744-w.

## Introduction

### Caring relatives and people with dementia

According to official figures, approximately 1.6 million people in Germany and over 55 million people worldwide are currently living with dementia (DAlzG 2020; Gauthier et al. 2021). In 2030, there will be over 2 million cases in Germany and approximately 78 million cases worldwide (Gauthier et al. 2021; DAlzG 2020). However, the exact figures are assumed to be even higher, as an unknown number of cases go unreported. In Germany, approximately two-thirds of people with dementia (PWD) are cared for by relatives at home (Federal Statistical Office of Germany 2018). This makes caring relatives (CRs) a mainstay of the German care system and demonstrates the relevance of their support (Blome et al. 2018).

Caring for a PWD at home can be particularly burdensome for CRs because of the symptoms associated with the disease such as changes in character and behaviour accompanying changes in relationships which occur, most of which are difficult to predict and are progressive (Frewer-Graumann 2020). This often has a negative impact on the health and daily lives of CRs (Kruse 2017; Rothgang and Müller 2018). Numerous studies indicate that support services can improve the well-being and quality of life of CRs of PWD (Safavi et al. 2019). The municipal communities with their stakeholders and local authorities such as communal actors, social workers, volunteers etc. are one entity responsible for offering adequate support for CRs. The *Seventh Report on the Elderly* published by the German Federal Government called on municipal communities to create opportunities for developing and implementing support and care for CRs (Blome et al. 2018; BMFSFJ 2016). According to Chapter 9, §71 of Book XII of the Social Code, the German municipal communities are responsible for the services in the public general interest and a local culture of care (Brettschneider 2020). However, because of divided competences between federal government, states, and municipal communities, the responsibilities vary between the different states, and the degree to which support is actually implemented also varies greatly between communities (BMG 2016; Holroyd-Leduc et al. 2017; Jensen et al. 2015). Frequently, care within the community is only carried out on a project basis, and long-term implementation fails (von Lützau-Hohlbein 2017).

### Implementation science and the Theoretical Domains Framework

For the successful realisation and long-term implementation of an (evidence-based) intervention into "standard care", adequate procedures are required in the implementation process (Greenhalgh et al. 2005; Hoben 2015). There are multiple strategies and theories for designing and controlling the implementation process, such as the “Consolidated Framework for Implementation Research” by Damschroder et al. (2009) or the “Theoretical Domains Framework” by Michie et al. (2005) (Grol et al. 2013; Hoben et al. 2015).

Discussions and research have shown that the level of support is highly dependent on individual actors in the communities (CAs) such as social workers, volunteers, nurses etc. (BMFSFJ 2016; Wittek et al. 2022a). In addition to structural determinants such as time or money, (personal) characteristics of CAs such as knowledge, professional roles, and motivation play an important role in implementing support services for CRs of PWD. The present article applies the Theoretical Domains Framework (TDF) as it addresses the implementation behaviour of different actors and their aforementioned characteristics (Michie et al. 2005). In a six-phase consensus process with the help of health psychology theorists, health psychologists and health services researchers, Michie et al. (2005) generated a validated theoretical framework for implementation science, the TDF (Michie et al. 2005). The application of the TDF is used to understand behavioural change processes, which are part of an implementation process. The TDF answers the question: *Which characteristics of actors influence their behaviour during the implementation of interventions?* and (the original version) consists of 12 domains, e.g., knowledge, beliefs about capabilities, or social influences (see Appendix Fig. 1).

This study adapted the domains of the TDF to the context of gerontology, municipal communities and demands of CRs of PWD. Furthermore, the adapted framework consists of 11 newly developed domains, and were compared to existing validated questionnaires, especially to the questionnaire of Huijg et al. (2014), tested and readjusted to the *Community Implementation Behaviour Questionnaire* (CIBQ) (see Appendix Table 1).

The idea behind the adaptation was to answer the question: *Which characteristics of CAs influence their behaviour during the implementation of support services for CRs of PWD in communities?* To ensure better clarity and understanding, only three out of 11 domains are considered in the following. Those three domains should be the most frequently addressed domains within the town hall talks.

**Fig. 1:**
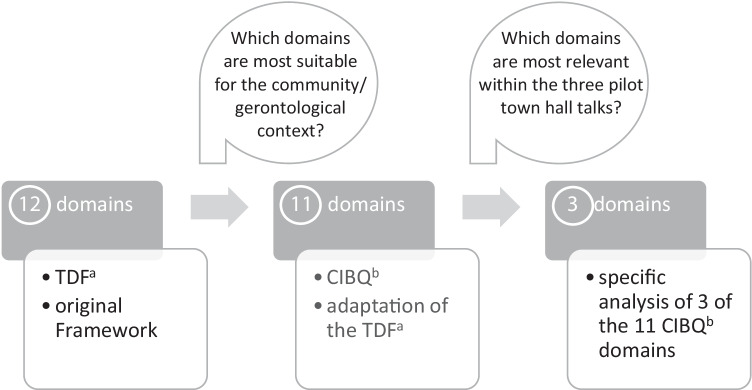
Adaption and selection process of the discussed domains. ^a^TDF = Theoretical Domains Framework; ^b^CIBQ = Community Implementation Behaviour Questionnaire

The adaptation and selection process of the domains discussed in this paper can be traced in Fig. 1.

### Town hall meetings

To design and develop the implementation of interventions, e.g., support services for CRs of PWD, participatory methods are recommended (Bergold and Thomas 2020). As described by Grol (1997) and Hoben et al. (2015), among others, designing and developing the implementation of interventions is the first step of implementation. It is called *planning and developing proposals for change to the current care situation* (Grol 1997; Hoben et al. 2015). As “a healthy democracy depends on the ability of citizens to affect the public policies that deeply influence their lives […]” (Lukensmeyer and Brigham 2002, p 351), one opportunity to facilitate the participation of target groups, citizens, and stakeholders is the method of town hall meetings. This style of public dialogue has a long tradition in the USA starting in the seventeenth century, and is an established part of the municipal participatory culture (Bipar 2016). In town hall meetings, members of different groups and functions have the opportunity to exchange ideas and learn about each other's needs (Bergold and Thomas 2020). While this method was originally used primarily in a political context, it is now applied in a wide variety of contexts, including medical care (Bipar 2016). Various studies show the added value of (digital) town hall meetings in different settings (Allen et al. 2020; Jayawardena et al. 2020; Wittek et al. 2022b). For example, different CAs could identify coronavirus pandemic-related gaps in supporting the CRs of PWD in the community through participation in town hall meetings (Wittek et al. 2022b). The benefits of participatory methods such as town hall meetings become clear in this context (Wiloth et al. 2020; Bergold and Thomas 2020).

This article wants to combine a town hall meeting in German municipal communities about the living situation of CRs of PWD and possible offers of support at the community level *with* the characteristics of CAs, which might be necessary for successful implementation of those support services (Wiloth et al. 2020). The scope of the town hall meetings is to create a dialogue between the CRs of PWD and actors of different fields of work in the community, such as municipal administration, health care, voluntary work, consulting, church, sports, culture, education, and housing. Through this dialogue structure, the various participants can influence both the content of the research project and the content of future care. This provides a participatory approach to the development of care. Within this participation, CAs have the opportunity to report on eventual existing services and reflect on their past and current implementation behaviour with regard to support services for CRs of PWD, as well as on their possible future behavioural changes. CAs should *listen and report, and reflect and act*. The participation of CRs consists of describing their daily lives with all their supportive measures or people and barriers or gaps in care. Furthermore, CRs should articulate their needs and how they can become part of optimising their own situation together with CAs and other CRs. Thus, CRs should *describe, wish, and get involved*. These events took place in the respective town halls of different communities in Germany or in a digital format because of the coronavirus pandemic. Interested citizens were invited to listen and to join the discussion. The aim of this public dialogue was to exchange information about needs, burdens and rewards of caring for PWD, as well as the use or needs of support services for CRs of PWD in their community.

### Aim and research questions

The aim of this study is to investigate (1) which of the previously presented and adapted domains appear in a public dialogue, and (2) the results that town hall meetings produce in terms of support services for the CRs of PWD.

## Materials and methods

Within the research project, "Giving a voice to caring relatives of persons with dementia — The Town Hall Project", data was collected, and in the current study this data was analysed on the basis of new research questions (see above). The methodological procedure of recruitment, data collection, and data analysis were published by Wiloth et al. (2020) and can be read in detail here. The Town Hall project received a positive vote from the Ethics Committee of the University of Heidelberg, Faculty of Behavioural and Cultural Studies, in 2019.^1^

### Data — town hall meetings

The town hall meetings described in the background section last 3 hours and are moderated by SW using predeveloped leading questions. The interview guide was developed according to the research questions and on the basis of theoretical concepts such as resilience (Ryff and Keyes 1995) and psychological well-being (Connor and Davidson 2003) and literature on the topics of vulnerability, stress, maturity, and resources, as well as on the basis of the contents of the *Sixth and Seventh Report on the Elderly* published by the German Federal Government (Kruse 2017; BMFSFJ 2016). Up to approximately ten CRs and approximately ten CAs participated in each talk. After approximately 6 weeks, each town hall meeting was followed by a 1-hour focus group. The different CAs took part in this focus group to reflect on the content of the previous event. These were moderated by SW using predeveloped leading questions as well, and took place in person or virtually, according to the pandemic situation.

The data were collected from November 2019 to January 2022. Based on defined criteria such as region (urban and rural), the number of inhabitants and accessibility, municipal communities were selected to be invited to participate in the project. For interested communities, preliminary meetings were held with representatives of the communities to provide detailed information on the background and the process of the project. A total of 45 municipal communities received an invitation to participate in the project. Sixteen municipalities participated and each had a town hall talk. Nonparticipation was due to difficulties in finding a date or the effort involved. To answer the aforementioned research questions, data from 14 out of 16 town hall meetings and focus groups were analysed. At the time of analysis, the other two town hall meetings and focus groups had not yet fully been analysed. The distribution and location of the 16 different municipal communities within Germany can be read elsewhere (Wiloth et al. 2020; Wittek et al. 2022b). The characteristics of *n* = 93 CRs and *n* = 138 CAs can be found in Tables 1 and 2.

**Table 1:** Characteristics of CRs and PWDs

Characteristics^a^ (*n* = 93)	Total^b^
Sex of CR	
Female	74.2 (69)
Male	25.8 (24)
Age of CR (in years)	65.69±11.36
Relationship between CR and PWD	
Wife/ husband/ partner	53.8 (50)
Daughter/ son	41.9 (39)
Daughter-/ son-in-law	1.1 (1)
Other	3.2 (3)
Education^c^ of CR	
Primary school	21.7 (20)
Secondary school	26.1(24)
High school graduation	10.9 (10)
University degree	25.0 (23)
PhD	5.4 (5)
Other	10.9 (10)
Occupation of CR	
Yes	34.4 (32)
No	65.6 (68)
Duration (months) of care of PWD by CR	50.86±40.03
Sex of PWD	
Female	52.2 (48)
Male	47.8 (44)
Age of PWD	80.17±8.16
Care level of PWD^d^	
No care level	1.1 (1)
Care level I	5.4 (5)
Care level II	17.2 (16)
Care level III	43.0 (40)
Care level IV	24.7 (23)
Care level V	8.6 (8)

^a^Data presented as percentage (number) except for: age of CR, duration of care of PWD by CR, age of PWD: these are presented as mean and standard deviation.

^b^Data were missing for education of CR(*n* = 1), sex of PWD (*n* = 1), age of PWD (*n* = 1)

^c^The education definition corresponds to the German education system and has been translated accordingly

^d^According to the national long-term care insurance scheme

**Table 2:** Characteristics of CAs

Characteristics^a^ (*n* = 138)	Total^b^
Sex of CA	
Female	66.1 (80)
Male	33.9 (41)
Age of CA	53.15±10.73
Education^c^ of CA	
Primary school	0.8 (1)
Secondary school	14.9 (18)
High school graduation	11.6 (14)
University degree	66.9 (81)
Other	5.8 (7)
Occupational field of CA	
Politics	8.0 (11)
Municipal administration	15.2 (21)
Consulting	13.0 (18)
Nursing	13.0 (18)
Medicine/ pharmacy	10.9 (15)
Church	9.4 (13)
Culture	2.2 (3)
Sports	7.2 (10)
Education	8.0 (11)
Living	4.3 (6)
Volunteering	8.7 (12)

^a^ Data presented as percentage (number) except for: age of CA: this is presented as mean and standard deviation.

^b^Data were missing for sex of CA (*n* = 17), age of CA (*n* = 16), education of CA (*n* = 17)

^c^The education corresponds to the German education system and has been translated accordingly

### Data analysis

The town hall meetings and focus groups were recorded, transcribed, and evaluated by means of qualitative content analysis based on Kuckartz (2018). For data analysis, MAXQDA 2020 (Verbi Software GmbH, Berlin) was used. To ensure reliability and validity, the analysis was performed by three coders in a consensual process (MW, AK, HV). Afterwards, the results were discussed by the project team. First, the project team created deductive codes according to the adapted domains. For a good understanding, only the three domains that occur most frequently in the first three (pilot) town hall meetings are considered in the following analysis.

Second, the project team created inductive codes while analysing the focus groups. Thus, the changes in CRs care caused by town hall meetings can be shown.

The entire research process was accompanied and documented by keeping a logbook (Rädiker and Kuckartz 2019).

## Results^2^

### Domains in public dialogues

As already mentioned, only three of the 11 domains (see Appendix Table 1) are considered in this study, to ensure better clarity and understanding. Table 3 shows the frequencies of the occurrence of domains within the first three (pilot) town hall meetings. The following three domains came up most frequently within those talks: *Knowledge, Goals, and Sociopolitical Context.* Further analysis points out that those domains show up in public dialogues in different ways. Each of the 14 town hall meetings thematises at least two out of the three domains.

**Table 3:** Number of occurrences of the domains within the first three town hall meetings

Domains		Number of Occurrences
D1	Knowledge	24^a^
D2	Skills	13
D3	Social/professional role and Identity	0
D4	Beliefes about capabilities	0
D5	Beliefes about consequences	0
D6	Goals	14^a^
D7	Sociopolitical context	25^a^
D8	Social influences	4
D9	Emotions	10
D10	Reinforcement	0
D11	Nature of the behaviour	7

^a^Most frequent occurred domains

### Knowledge

The CAs of each town hall meeting share their knowledge of CRs of PWD or rather the care situation in Germany. CAs from communities 1, 2, and 3 also have passages where they express not knowing enough about the target group and its circumstances. Overall, different aspects were discussed:

Some CAs show their knowledge during the town hall meetings about the demographic situation and about the demographic change: “*So when there are fewer and fewer relatives who can provide care, when the situation changes, other things have to take effect*.” (4) In addition, their awareness of the relevance and necessity of CRs of PWD becomes clear within the data: “*They are the biggest care service we have in the country.*”(4) and “*Without you, without the relatives, our system would completely collapse.”*(10)

The CAs not only know about the care situation in Germany but also about the individual situation of the CRs of PWD. They have themselves partially experienced what it is like to accompany a relative with dementia. The following topics are raised during the town hall talks: lack of support, missing money, many services that need to be paid by the families themselves, overload of CRs, or the lack of flexibility of support services: “*[…] however structures, flexible structures have to be created that relieve the caring relatives regularly and also a bit more extensively than once a month or so, so that they can still live their own lives a bit.*”(4) Furthermore, CAs talk about their knowledge and experiences of courage, which is sometimes needed to get help and to accept help as a CR: “*[…] I noticed […] that there is an incredible inhibition threshold for relatives to "come out", because embarrassing things happen at home.”*(7)

On the other hand, some CAs did not know the high number of PWD or rather the development of the disease. They also mentioned not knowing about some of the existing services: “*[…] I myself was not so aware that the [population with dementia] is so large, has such a weight and corresponding importance for our city.*”(2)

### Goals

Concrete goals are not discussed in each town hall talk. The CAs of communities 5 and 14 did not mention any goals, and in some communities, only ideas or ventures were shared. Each of the shared goals had a different focus.

During the town hall meetings, the CAs talked about creating and strengthening networks with colleagues or other CAs. They mentioned that they wanted to become mediators or multipliers to facilitate the exchange of information about support opportunities with the CRs of PWD. While they themselves are willing to become more attentive to the target group, they also want to motivate colleagues to do so: “*And I think that it is of course also a medical task to instruct the relatives at an early stage […]. I will also take this with me to our doctors' meeting, we regularly exchange ideas that we should take the time even more intensively, especially in the case of early forms of dementia.*”(1)

In addition, CAs talked about engaging the public. They have goals such as organising public events to raise the awareness for CRs and PWD and doing public relations: “*We have already said that we would like to go more public and that is also one of our goals, to appear regularly in the press with topics that move the citizens and our clients.*”(8)

New support services and, above all, affordable support services are further goals which were mentioned within the town hall meetings, especially because of the inflexible, limited, and bureaucratic way of getting support services reimbursed: “*The neighbourhood assistance no longer exists in the way we had. We are in the process of rebuilding it, which unfortunately is not that easy and we need a lot of patience. However, that is our goal.*”(4)

### Sociopolitical context

The CAs of each town hall meeting reported on the sociopolitical context and the connection to CRs of PWD and their situation. Except for communities 6 and 10, the CAs of each community shared positive and negative aspects of the sociopolitical context. In communities 3, 11, and 12, negative points predominated. Overall, different aspects were discussed.

When talking about the sociopolitical context of implementing support services of CRs of PWD, CAs discussed positive and negative aspects. It was often mentioned that resources are scarce for developing the necessary support services for CRs of PWD possible. Resources are mainly time, money, and human resources. Networks and groups which might be partly already established could help out: “*Well, the structures are prescribed by the long-term care insurance. That is, where you finance the services […].*”(14)

Furthermore, CAs said that federalism as well as the separated responsibilities between city, municipal community, and district complicate the implementation of support services for CRs of PWD. This fact also aggravates using the services and makes it more bureaucratic: “*One can either like or dislike the fact that there are responsibilities with the city, some with the district, and some with the payers. That does not make it easy for the carers […]. Too much bureaucracy, too many phone calls back and forth.*”(12) After the CAs, another aspect within the sociopolitical context is laws and regulations, which are very strict and not very supportive for the care sector: “*[…] that short-term care is so reluctant to be offered for economic reasons. I find it distressing and it sheds a terrible light on our social system here.*”(11)

During the town hall talks, some CAs say that those in politics need much more awareness of care, CRs, and PWD. This might succeed through founding organisations supporting the opinion, interests, and needs of the target group in political decisions: “*Relatives' organisations that can also act as lobbyists, and only if everyone from all directions, so to speak, carries this upwards again and again, will there also be an awareness of this.*”(8)

### Support and care services for CRs of PWD

**Fig. 2:**
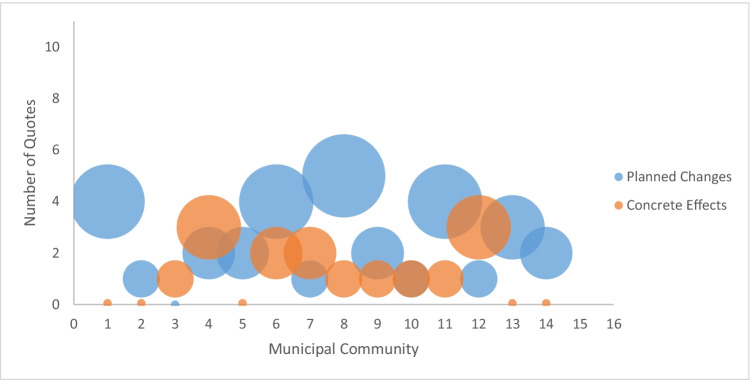
Number of quotes per municipal community. *The size of the bubbles reflects the number of the quotes on the *y*-axis and the municipal communities on the *x*-axis. **The data from the communities 15 and 16 have not yet been fully analysed

Looking at the focus groups of the respective municipal communities and their CAs, which took place approximately 6 weeks after the town hall meetings, changes in the support services for CRs of PWD can be seen. Not every municipal community reported concrete effects that have already been implemented, but there are CAs who at least have already planned some changes or optimisations for the support of the target group in each community (see Fig. 2).

Figure 2 shows the number of quotes (*y*-axis) concerning *planned changes* (blue bubble) and *concrete effects* (orange bubbles) per municipal community (*x*-axis). The size of the bubbles indicates the number of quotes in each case.

During the focus groups, the CAs mentioned so-called *concrete effects* as well as *planned changes* to support the CRs of PWD in their community. As the categories are quite similar, the quotes and contents are reported together.

Among other things, they talked about offering (further) possibilities to inform CRs about themes such as care, dementia or support services. This could be in the form of brochures: “*[…] we used the time to create a special dementia guidebook.*”(3), events, flyers, homepages, or lectures: “*[…] we wanted to organise a lecture in the summer […] about dementia. Just basics again. Which is public and takes place here in the town hall […].*”(1)

In addition, CAs intend to organise different services for CRs and/or PWD. Some services can be used exclusively by CRs to spend time for themselves, to recharge or just to free their minds from everyday tasks and responsibilities. This could be a yoga session, a painting course, or a discussion group because CRs “*[…] need to be able to exchange experiences with other like-minded people. In fact, this has led us to start another group, which is there for the exchange of experiences.*”(7)

Other offers are addressed exclusively for PWD, where CRs gain time, for example, to get things done. These are services such as daycare or similar care offers: “*There will be daycare on Saturdays once a month on a trial basis for 6 months next year. Depending on how this is accepted, it can be continued.*”(6) Then, there are services for both CRs and PWD. Both can spend time together outside of their everyday lives: “*We want to set up a kind of sensory garden where relatives can sit down with the person they are caring for without having to worry.*”(14) Sometimes there is also a professional who looks after them and who is caring for both of them. CAs know about the need for flexibility, affordability, and practicability of such offers. That is why they are thinking along of appropriate time and place: “*We are now planning to create an offer that takes place at times when working people can also come along.*”(4)

In some communities, human resources are needed to plan, organise, and offer support services, or rather to have someone who knows about the possibilities within the community and who can consult the CRs of PWD. For this reason, the CAs are talking about hiring somebody: “*[…] neighbourhood assistance was also mentioned in the town hall talk, and we have developed a concept. We have also promised a position.*”(4)

“*We have already set up a mailing list. There is a new e-mail address […] where you can contact us to get on the mailing list or to ask questions about dementia in general.*”(8) This quote is one example of the networking effect, the town hall meeting had. The CAs talked in the focus groups about getting in touch with CAs and CRs they did not know before. They report on exchanged contact details, meetings and newly formed working groups: “*Following the town hall talk, we networked very intensively: District Office, Care Support centre and the City […].*”(12)

Raising awareness of the general public as well as the policy-makers is another point the CAs talked about during the focus groups. For this purpose, they “*[…] are going to bring it to a municipal council committee right away and [we] are going to call for municipal political support there as well.*”(5) In addition, they plan to involve citizens by organising public events about care and dementia or arranging for the dementia network to have its own newspaper column: “*[…] there [in the newspaper] we are actually allowed to present ourselves once a month with the headline "News from the Dementia Network" and with our logo.*”(8)

## Discussion

The article examined the benefit of CAs thematising three domains from the TDF within a public dialogue for the improvement of the care of CRs of PWD. Only these three domains were part of the presented results. Above all, the data showed that most CAs are aware of the themes along with the domains of *knowledge*, *goals*, and *sociopolitical context* and brought them up themselves during town hall meetings. According to the scope of the topics discussed at the meetings, the focus groups of the municipal communities show varying effects. The first mentioned domain *knowledge* described whether the participating CAs are aware of the situation and relevance of CRs and PWD. Their statements in the town hall meetings show that in each community, there are at least some CAs who bring this knowledge up. On the other hand, there are also a few CAs who communicate their lack of knowledge. In those communities (1, 2, 3), less concrete effects were reported in the focus groups (see Fig. 2). Within the second domain, *goals,* a distinction must be made between concrete goals and more generally formulated goals. In two communities (5, 14), there were no goals mentioned, and they also reported no concrete effects in the focus groups (see Fig. 2). The third domain, *sociopolitical context,* is often addressed negatively. When the negative comments outweigh the positive ones, this mostly leads to less or no concrete outcome in the focus groups.

**Fig. 3.**
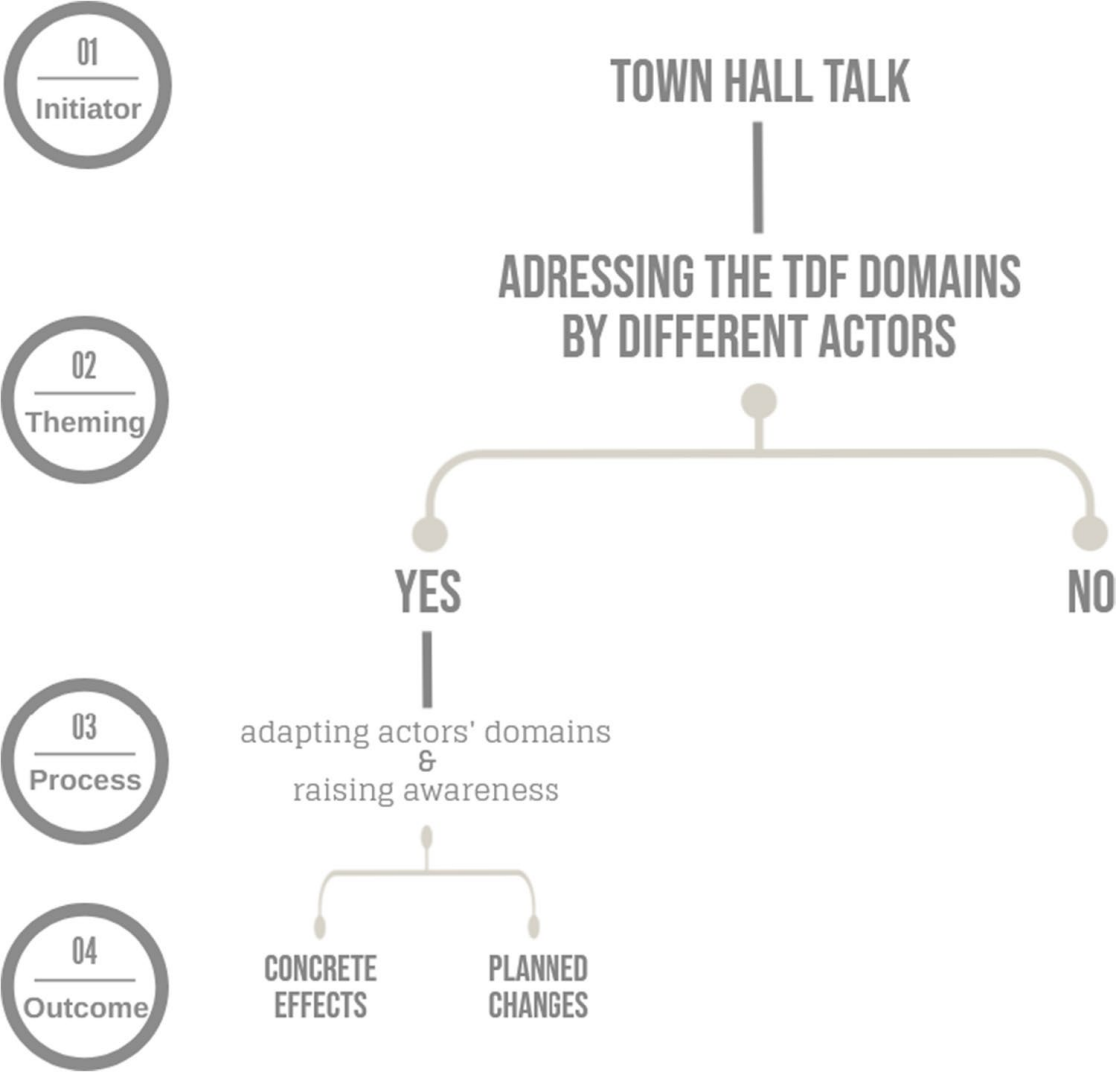
Illustration of the relationship between the town hall meetings, the TDF domains, and the resulting effects

The results indicate that the intensity of the upcoming domains within the town hall meetings influences the CAs and their statements regarding the improvement of support services for the CRs of PWD (see Fig. 2). The data show that communities that have *less* knowledge, *fewer* goals or *more* *negative experiences* with the sociopolitical sector reported *fewer concrete effects* within the focus groups. This could be a first indication of a link between the presence of the TDF domains and an improvement of the support for CRs of PWD. This link leads to the assumption that thematising TDF domains in a public dialogue can be seen as the first step in the implementation of support services (see Fig. 3).

With regard to the procedure in the implementation process, among others Grol (1997) and Hoben et al. (2015) describe this step as *planning and developing proposals for change for the current care situation*. Those CAs who reported concrete effects and solutions are already well advanced in the implementation process. Those CAs who have ideas or plan changes need to follow the next necessary steps: 1) identification of facilitating and hindering factors for implementation, 2) application of interventions to overcome these barriers, 3) planning of implementation, and 4) steering and evaluation of implementation (Grol 1997; Hoben et al. 2015).

With regard to one community, the CAs who thematised a domain within a town hall meeting might not be the same persons who developed support for CRs reported within the focus group. The question arises as to what extent the domains that are addressed by one CA are related to an effect reported by another CA. The findings reflect the consensus in the current literature that raising community awareness and understanding, for example, of dementia, enables communities to act, e.g., to develop dementia-friendly communities (Buckner et al. 2019; Phillipson et al. 2019; Williamson 2016). Creating awareness through conversation, as has been shown in this paper, is also considered an effective method in the literature (Buckner et al. 2019; Phillipson et al. 2019). However, conversation is not the only way to raise awareness and knowledge. Media, educational events, lectures, or information leaflets are also well-known techniques within the current research (Buckner et al. 2019; Heward et al. 2017; Phillipson et al. 2019; Williamson 2016). The fact that some actors report from their own professional or personal experience and that family carers also take part in town hall meetings supports the findings that first-hand experiences in particular lead to awareness changes (Hung et al. 2021; Williamson 2016).

As our method is participatory by involving different stakeholders, practice partners, and CRs, it is also supported by the results of Dorant and Krieger (2017). They involved service providers as co-researchers to foster awareness and motivation. In addition, Williamson (2016) and Heward et al. (2017) state that it is important to raise awareness within different professions or fields of work, such as sports clubs, volunteering, and policy-making, as is also done in our study format. The participation of different CAs and CRs of PWD can thus achieve what has already been described in the background section. While CAs *listen and report*, and *reflect and act*, or develop an awareness of CRs and the necessary support services, CRs of PWD *describe, wish* and *get involved* to shape their future as well as future support together with the communities.

### Strengths and limitations

The strengths of this paper include the link between strategies or methods of implementation science and the project itself with its given data. In addition, the use of the TDF as a theoretical foundation of qualitative analysis needs to be rated positively because this framework has already proven its usefulness in different contexts (Huijg et al. 2014; Murphy et al. 2014; Seward et al. 2017). However, some methodological limitations may have affected the strength of evidence or rather informative value: no control group was considered. Accordingly, it is not possible to make a statement about what effects would have arisen without the town hall meetings. Nevertheless, the actors and stakeholders from the community mentioned that they follow the results and effects because of the public event. Another limitation consists of the heterogeneity of the reported effects. It is difficult to compare, for example, a new contact or flyer with realising a new position for senior citizen counselling. The time span in which the focus group took place could also have influenced the results. It could have been too long, so that the participants could not remember the content of the town hall talks, or it could have been too short to achieve results. In addition, the analysis of the data from the last two communities (15 & 16) had not yet been completed. The aspect of presenting only three of 11 domains provides more clarity, but does not give any insight into the remaining eight domains and how they are addressed in the town hall talks.

## Conclusion

The present paper highlights the beneficial application of the TDF within a public dialogue on improving care for CRs of PWD. As stated in the literature, creating awareness might be central to this finding. The first indications become clear; that addressing at least the three TDF domains of *knowledge, goals* and *sociopolitical context* encourages actors and stakeholders to find solutions for the deficiencies in the care of CRs of PWD in their communities. Accordingly, in future discussions with actors, a targeted approach to the domains can be made to build awareness to achieve the effects described. Since the domains were not developed exclusively for the outlined context, this approach can also be applied to other care topics. As a result, the care of CRs and other target groups can be optimised. For future research, it would be beneficial to apply the described domains of the gerontological context in a resource-efficient, time-saving and generalisable way, as is possible with a quantitative questionnaire.

## Supplementary information

ESM 1 (PDF 657 kb)

## Data Availability

The data is available on request by the corresponding author.

## Footnotes

1 In February 2021, due to the Corona pandemic, an amendment was submitted to the Ethics Committee, which was again positively assessed on 24.02.2021 (2021 1/1-A1).

2 As the project was carried out in Germany, the following quotations in the Results section are literal translations from German. The original German quotations can be found in Appendix Table 2.

## Code availability

The code is available on request by the corresponding author.
