# Supplementary material for: Community support for caring relatives of people with dementia: qualitative analysis using the Theoretical Domains Framework
Source: Z Gesundh Wiss. 2022 Aug 12:1–11. Online ahead of print. doi: 10.1007/s10389-022-01744-w (PMC9371957; doi:10.1007/s10389-022-01744-w)
Supplement: Supplementary file 1 — (PDF 657 kb) [file 10389_2022_1744_MOESM1_ESM.pdf]

Supplementary information

12 domains of the „Theoretical Domains Framework“:

1. Knowledge
2. Skills
3. Social/professional role/identity
4. Beliefs about capabilities
5. Beliefs about consequences
6. Motivation & goals
7. Memory/ attention/ decision process
8. Environmental context & resources
9. Social influenses
10. Emotional regulation
11. Behavioural regulation

Fig. 1 Domains of the Theoretical Domains Framework (Michie et al. 2005)

Tab. 1 Definition of the domains and the adoption to the gerontological context

	Domain	Original Definition	Adaptation to the Gerontological Context
**D1**	**Knowledge**	An awareness of the existence of something.	The CAs have the knowledge about the situation and understand the relevance of CRs of PWD.
**D2**	**Skills**	An ability or proficiency acquired through practice.	The CAs have the skills and training to implement support services for CRs of PWD in the community.
**D3**	**Social/Professional Role and Identity**	A coherent set of behaviours and displayed personal qualities of an individual in a social or work setting.	The behavioural spectrum and the personal qualities of the CAs with regard to implementing support services for CRs of PWD in the community are part of the professional setting.
**D4**	**Beliefs about Capabilities**	Acceptance of the truth, reality or validity about an ability, talent, or faculty that a person can put to constructive use.	The CAs are confident in their abilities to implement support services for CRs of PWD in the community.
**D5**	**Beliefs about Consequences**	Acceptance of the truth, reality or validity about outcomes of a behaviour in a given situation.	The CAs understand the advantages and disadvantages of implementing support services for CRs of PWD in the community.
**D6**	**Goals**	Mental representations of outcomes or end states that an individual wants to achieve.	The CAs have goals that they would like to achieve regarding implementation of support services for CRs of PWD in the community.
**D7**	**Sociopolitical Context**	Any characteristics of the sociopolitical context that discourages or encourages the development of skills and abilities, independence, social competence, and adaptive behaviour.	The sociopolitical context has characteristics that motivate or discourage CAs to develop competences, skills, adaptive behaviours and independence regarding the implementation of support services for CRs of PWD in the community.
**D8**	**Social Influences**	Interpersonal processes that can cause individuals to change their thoughts, feelings, or behaviour.	Interpersonal processes that lead to a change in the CAs' thoughts, feelings or actions regarding the implementation of support services for CRs of PWD in the community.
**D9**	**Emotions**	A complex positive/negative reaction pattern involving experiential, behavioural, and physiological elements by which the individual attempts to deal with a personally significant matter or event.	The CAs have positive emotions about implementing support services for CRs of PWD in the community.
**D10**	**Reinforcement**	Increasing the probability of a response by arranging a dependent relationship, or contingency, between the response and a given stimulus.	A given stimulus increases the probability of CAs implementing support services for CRs of PWD in the community.
**D11**	**Nature of the Behaviour**	The nature of the aggregate of all responses made by an individual in any situation.	The CAs have an original (intuitive, personal) way of behaving on which all actions and reactions in relation to CRs of PWD are based.

*Domain definitions were based on definitions from Huijg et al. (2014).

Tab. 2 Original German quotes and the literal English translation

Original German quotes	Literal English translation
Knowledge	
“Wenn es also immer weniger Angehörige gibt, die pflegen können, die Situation sich verändert, dann müssen andere Dinge greifen.“(4)	“So when there are fewer and fewer relatives who can provide care, when the situation changes, other things have to take effect.”(4)
“Sie sind der größte Pflegedienst, den wir hier überhaupt im Land haben“(4)	“They are the biggest care service we have in the country.”(4)
„Ohne Sie, also ohne die Angehörigen würde unser System völlig zusammenbrechen.“(10)	“Without you, without the relatives, our system would completely collapse.”(10)
„[…] dass wie auch immer Strukturen, flexible Strukturen geschaffen werden müssen, die die pflegenden Angehörigen regelmäßig und auch ein bisschen umfangreicher als einmal im Monat oder so entlasten, damit diejenigen auch noch ein bisschen ihr eigenes Leben leben können.“(4)	“[…] however structures, flexible structures have to be created that relieve the caring relatives regularly and also a bit more extensively than once a month or so, so that they can still live their own lives a bit.”(4)
“[…] ich habe […] gemerkt, dass es eine unglaubliche Hemmschwelle gibt für Angehörige sich zu "outen", ne, weil da peinliche Sachen zuhause passieren.“(7)	“[…] I noticed […] that there is an incredible inhibition threshold for relatives to "come out", because embarrassing things happen at home.”(7)
„[…] mir war das selbst nicht so bewusst, dass das [Bevölkerung mit Demenz] doch so eine große Anzahl hat, so das Gewicht hat und dann auch entsprechend Bedeutung für auch unsere Stadt.“(2)	“[…] I myself was not so aware that the [population with dementia] is so large, has such a weight and corresponding importance for our city.”(2)
Goal	
„Und ich denke, das ist natürlich auch eine ärztliche Aufgabe, die Angehörigen dann frühzeitig zu instruieren […]. Das werde ich auch hier in unsere Ärzterunde mitnehmen, wir tauschen uns ja regelmäßig aus, dass wir da gerade bei Frühformen der Demenz noch intensiver uns die Zeit nehmen sollten.“(1)	“And I think that it is of course also a medical task to instruct the relatives at an early stage […]. I will also take this with me to our doctors' meeting, we regularly exchange ideas that we should take the time even more intensively, especially in the case of early forms of dementia.”(1)
“Wir hatten ja schon gesagt, dass wir mehr an die Öffentlichkeit gehen möchten und das ist auch ein Ziel von uns, regelmäßig in der Presse zu erscheinen mit Themen, die die Bürger und auch unsere Klienten bewegt.“(8)	“We have already said that we would like to go more public and that is also one of our goals, to appear regularly in the press with topics that move the citizens and our clients.”(8)
„Die Nachbarschaftshilfe, die es nicht mehr gibt in dieser Art, wie wir sie hatten. Da sind wir dabei, sie neu aufzubauen, was leider auch nicht so einfach ist und da brauchen auch wir einen langen Atem. Aber das ist unser Ziel.”(4)	“The neighbourhood assistance, no longer exists in the way we had. We are in the process of rebuilding it, which unfortunately is not that easy and we need a lot of patience. However, that is our goal.”(4)
Socio-political context	
“Also die Strukturen sind ja von der Pflegeversicherung vorgegeben. Also dort, wo Sie die Angebote finanzieren […]”(14)	“Well, the structures are prescribed by the long-term care insurance. That is, where you finance the services […].”(14)
„Das kann man jetzt gut oder weniger gut finden, dass es eben Zuständigkeiten bei der Stadt gibt, ein paar beim Landkreis, ein paar wiederum bei den Kostenträgern. Das macht es den Pflegenden nicht einfach, das höre ich jetzt auch hier bei Ihnen immer wieder raus. Zu viel Bürokratie, zu viel hin und her telefonieren.” (12)	“One can either like or dislike the fact that there are responsibilities with the city, some with the district, and some with the payers. That does not make it easy for the carers […]. Too much bureaucracy, too many phone calls back and forth.”(12)

"[…]dass aus wirtschaftlichen Gründen Kurzzeitpflege so ungerne angeboten werden. Ich finde es erschütternd und wirft ein schreckliches Licht hier auf unser Sozialsystem." (11)	"[…] that short-term care is so reluctant to be offered for economic reasons. I find it distressing and it sheds a terrible light on our social system here."(11)
"Angehörigen-Organisationen, die sich als Lobbyisten auch betätigen können und nur wenn alle aus allen Richtungen sozusagen das immer wieder nach oben tragen, entsteht da auch ein Bewusstsein dafür."(8)	"Relatives' organisations that can also act as lobbyists and only if everyone from all directions, so to speak, carries this upwards again and again, will there also be an awareness of this."(8)
concrete effects & planned changes	
„Wir haben […] die Zeit genutzt, um […] einen speziellen Demenz-Wegweiser zu erstellen."(3)	"[…] we used the time to create a special dementia guidebook."(3)
„wir wollten im Sommer einen Vortrag organisieren […] über Demenz. Einfach noch einmal Grundlagen. Der öffentlich ist und der hier im Rathaus stattfindet […]."(1)	"[…] we wanted to organise a lecture in the summer […] about dementia. Just basics again. Which is public and takes place here in the town hall […]."(1)
"[…] dieser Bedarf, sich mit anderen Gleichgesinnten austauschen zu können. Hat tatsächlich dazu geführt, dass bei uns auch noch eine weitere Gruppe an den Start geht, die für den Austausch da ist."(7)	"[…] need to be able to exchange experiences with other like-minded people. In fact, this has led us to start another group, which is there for the exchange of experiences."(7)
"Es wird im nächsten Jahr probeweise ein halbes Jahr lang 1x/Monat eine Tagespflege samstags geben. Je nachdem, wie das angenommen wird, kann es fortgesetzt werden."(6)	"There will be daycare on Saturdays for 1x/month on a trial basis for six months next year. Depending on how this is accepted, it can be continued."(6)
"Da wollen wir eine Art Sinnesgarten einrichten, wo man dann eben auch einmal Angehörigen die Möglichkeit gibt, sich hier bei uns hinzusetzen mit dem Menschen, den er versorgt, ohne dass er Sorge haben muss."(14)	"We want to set up a kind of sensory garden where relatives can sit down with the person they are caring for without having to worry."(14)
„Wir sind da jetzt dran an der Planung, ein Angebot zu schaffen, was zu Zeiten stattfindet, wo auch Berufstätige mitkommen könnten."(4)	"We are now planning to create an offer that takes place at times when working people can also come along."(4)
"[…] also im Rathausgespräch wurde ja auch Nachbarschaftshilfe angesprochen und da haben wir ein Konzept erarbeitet. Wir haben auch die Zusage für eine Stelle."(4)	"[…] neighbourhood assistance was also mentioned in the town hall talk, and we have developed a concept. We have also promised a position."(4)
"Wir haben jetzt schon eine Mailingliste aufgesetzt noch mal. Es gibt die neue E-Mail-Adresse […] wo man sich hinwenden kann, um eben auf die Mailingliste zu kommen beziehungsweise auch generell Fragen um das Thema Demenz stellen zu können."(8)	"We have already set up a mailing list. There is a new e-mail address […] where you can contact us to get on the mailing list or to ask questions about dementia in general."(8)
"Im Nachgang zu dem Rathausgespräch haben wir uns sehr intensiv vernetzt: Landratsamt, Pflegestützpunkt und Stadt […]."(12)	Following the town hall talk, we networked very intensively: District Office, Care Support centre and the City […]."(12)
„[…] werden wir gleich in ein gemeinderätliches Gremium bringen und dort uns auch eine kommunalpolitische Unterstützung holen."(5)	"[…] are going to bring it to a municipal council committee right away and [we] are going to call for municipal political support there as well."(5)
„[…] da [Zeitung] dürfen wir tatsächlich einmal im Monat uns auch präsentieren mit der Überschrift „Neues aus dem Netzwerk Demenz" und mit unserem Logo."(8)	"[…] there [newspaper] we are actually allowed to present ourselves once a month with the headline "News from the Dementia Network" and with our logo."(8)
